# Medication-Taking Habit and Outcome of Glucosamine Sulfate for Osteoarthritis Patients Influenced by National Health Insurance Regulations in Taiwan

**DOI:** 10.3390/jcm8101734

**Published:** 2019-10-19

**Authors:** Chia-Hao Hsu, Nin-Chieh Hsu, Chia-Lung Shih, Hsuan-Ti Huang, Chung-Hwan Chen, Pei-Hsi Chou

**Affiliations:** 1 Graduate Institute of Clinical Medicine, College of Medicine, Kaohsiung Medical University, No.100, Shiquan 1st Rd., Sanmin Dist., Kaohsiung City 80708, Taiwan; ecowarrior.tw@yahoo.com.tw; 2 Department of Orthopedics, College of Medicine, Kaohsiung Medical University, No.100, Shiquan 1st Rd., Sanmin Dist., Kaohsiung City 80708, Taiwan; hthuang@kmu.edu.tw (H.-T.H.); hwan@kmu.edu.tw (C.-H.C.); 3 Division of Adult Reconstruction Surgery, Department of Orthopedics, Kaohsiung Medical University Hospital, No.100, Tzyou 1st Rd., Sanmin Dist., Kaohsiung City 80756, Taiwan; stone770116@gmail.com; 4 Department of Orthopedics, Kaohsiung Municipal Ta-Tung Hospital, No.68, Jhonghua 3rd Rd, Cianjin District, Kaohsiung City 80145, Taiwan; 5 Department of Internal Medicine, National Taiwan University Hospital, No.7, Zhongshan S. Rd., ZhongzhengDist., Taipei City 10002, Taiwan; oeoeomorrison@yahoo.com.tw

**Keywords:** medication-taking habit, glucosamine sulfate, osteoarthritis, total joint arthroplasty, national health insurance regulations

## Abstract

This study compared the dosage and different medication-taking habits of glucosamine sulfate (GS) for osteoarthritis patients and evaluated the influence of the National Health Insurance (NHI) prescription guidelines. The subjects were collected from the Taiwan NHI Research Database from 1 January 2004, to 31 December 2008, and 10,501 osteoarthritis patients were included. Then, 271 patients who continuously used nonsteroidal anti-inflammatory drug (NSAIDs) and started to receive glucosamine for the first time since 2005 (no glucosamine use in 2004) were compared with 593 age-matched patients who continuously used NSAIDs but never received any glucosamine drugs from 2004 to 2008. The mean treatment duration of the glucosamine-treated and NSAID-treated groups was 40.38 ± 7.89 and 45.82 ± 3.89 months, respectively. The most common medication-taking habit was 250 mg 3 times a day for 3 months and discontinued for 3 months. It was as indicated and covered by the NHI. Only 0.7% of patients used the recommended daily dosage of 1500 mg. Patients using GS surprisingly had a higher incidence rate of joint replacement surgery than those who did not use GS. The NHI prescription guidelines may cause patient selection bias, which decreases the efficacy of GS. Moreover, patients tend to have an altered medication-taking habit, with a daily dosage of 750 mg, which is lower than the recommended therapeutic dose.

## 1. Introduction

Osteoarthritis (OA) is a degenerative joint disease which causes joint pain and limited function. Nonsteroidal anti-inflammatory drugs (NSAIDs) are the most common medications to relieve pain in OA [1,2,3]. Glucosamine sulfate (GS), which is also commonly used to relieve symptoms of OA, is thought to be safe. GS stimulates proteoglycan synthesis by chondrocytes. However, its efficacy in OA treatment still remains controversial [4,5,6,7,8,9,10,11,12,13].

The prescription of GS was covered and regulated by the National Health Insurance (NHI) in Taiwan before October 2018 (Taiwan’s health government finally cancelled the NHI coverage of GS since October 2018 due to the controversial and uncertain efficacy of GS). The NHI regulation may have an impact on physician prescription patterns. The utilization of GS may also be influenced by the patient’s willingness to pay if it is not covered under the NHI guidelines. Therefore, the efficacy of GS in Taiwan may be different compared with the results of the other countries.

In this study, we used 5year nationwide population-based data from Taiwan to evaluate the dosage and different medication-taking habits of GS and influence of the NHI prescription guidelines.

## 2. Materials and Methods

### 2.1. Data Sources

This study was approved by the Bureau of National Health Insurance of Taiwan (approval number: 99159). Data were collected from the National Health Insurance Research Database (NHIRD) from 1 January 2004 to 31 December 2008. Data from the last10 years were not selected. The first reason for this is that GS was no longer covered by the NHI since October 2018. Since then, no medication-taking data on GS could be found in the NHIRD. Another reason is that GS sales and consumption decreased gradually, and it became less popular in the last 10 years in Taiwan.

The NHIRD includes comprehensive information of the patients’ demographic data, diagnostic codes, details of prescriptions and medication codes, procedures/surgeries, dates of clinical visits and hospitalizations, and length of hospital stay, which were for research purposes only. This dataset was randomized using millions sampling distribution from a total of approximately 23 million people with comprehensive data on all the medications of 1 million people. Then, all medical records were followed from 2004 to 2008.

### 2.2. Study Population

Patients diagnosed with osteoarthritis as ICD-9-CM code 715, classified by the International Classification of Disease (ICD) coding system, were included in the study. Cases from Chinese medicine clinics (case type code: 11–14, 16, 19) and dental clinics (case type code: 21–29) were excluded. A total of 10,501 patients with OA were included. In standard and general clinical practice, only the radiographically confirmed OA is identified as OA.

### 2.3. Prescription Guidelines of GS Covered by Taiwan’s NHI (before October 2018)

Reimbursement of the GS cost should meet the following criteria based on the NHI regulations:

(1) Age ≥ 60 years;

(2) Ahlbäck classification of severity of knee OA [14]: ≤ stage III;

(3) OA symptoms ≥ 6 months;

(4) Minimum symptom severity was ensured by using the Lequesne’s severity index for knee OA [15]: at least 7 points.

Other additional instructions:

Only the maximum dosage of 750 mg per day is covered by the NHI. If a higher dosage (>750 mg) is necessary, applications for medical claims review should be made and approved before requesting prescriptions. The maximum duration of each GS treatment is 3 months. If there is no symptom improvement, the treatment should be stopped immediately. However, if the symptoms improved, GS treatment must be discontinued for the next 3 months after finishing a 3 month treatment course. Thus, the maximum treatment period was 2 courses of a 3month treatment in a year.

### 2.4. Study Design

Data were analyzed according to the following: (1) the medication-taking habit of patients with OA, (2) medication-taking habit of GS in patients with OA, and (3) incidence of primary total joint arthroplasty (TJA).

The Anatomical Therapeutic Chemical (ATC) Classification System, which was established by the World Health Organization, was used to assess the prescription drugs and frequency to investigate the medication-taking habit of patients with OA. The ATC code H02 stands for corticosteroid for systemic use, M01AX05 for glucosamine, N02BE01 for paracetamol, N02A for natural opium alkaloids, and M01A for anti-inflammatory and antirheumatic products, non-steroids (NSAIDs).

There are 3 commonly sold forms of glucosamine: (1) N-acetyl glucosamine, (2) Glucosamine hydrochloride (HCl), and (3) GS. Among them, only GS has been given a “likely effective” rating and was approved for treating osteoarthritis and covered by the NHI. Other forms of glucosamine were listed as dietary supplements and not approved as medical drugs. Therefore, all ATC codes with M01AX05 in the NHIRD stand for GS.

The inclusion criteria of the GS-treated group included patients with OA who continuously used NSAID and began to use GS since 2005 for the first time. The exclusion criteria of the GS-treated group were that patients used GS in 2004 in the database. According to the GS prescription pattern, patients were classified into the GS-treated and NSAID-treated groups. The GS-treated group consisted of 271 patients with OA who continuously used NSAID and began to use GS since 2005 for the first time. All patients who used GS in 2004 in the database were excluded because no data before 2004 were found. Furthermore, these patients may have been using GS for many years. Thus, it could lead to an unsuitable group comparison and may cause an incorrect outcome evaluation. However, if strict selection criteria were applied in selecting only patients who began to use GS for the first time since 2005, the sample size was limited and decreased to only 271 patients. For a robust scientific comparison, we have to accept a smaller sample size from a relatively large cohort.

A total of 7195 patients with OA who continuously used NSAID but never received any glucosamine from 2004 to 2008 were noted. We randomly selected patients from layered age groups in a case:control ratio of 1:2 to form an age-matched control group. The NSAID-treated group was comprised of 593 age-matched patients who continuously used NSAID but never received any glucosamine treatment from 2004 to 2008 in the database.

### 2.5. Specific Study End Point

TJA is generally the most common surgery for patients with advanced or end-stage OA. It represents a specific end point of OA treatment. Although partial joint replacement may be indicated in some selected patients, it was not popular and was rarely performed in Taiwan more than 10 years ago. Other surgery types for OA, such as high tibial osteotomy or arthroscopy, are not suitable to represent the end point of OA treatment. None of the patients in our study and control groups underwent partial joint replacement or other kinds of surgery for OA.

The follow-up time was started on the date wherein the study group first took GS in 2005 and on the date wherein the control group first took NSAID in 2005. The end of follow-up time was defined as the time that the patient underwent TJA. If the patient did not undergo TJA from 2005 to 2008, the end of the follow-up time was 31 December 2008.

### 2.6. Medication-Taking Habits of GS

The daily dosage was counted for each GS-treated patient and ranged from 250 to 2250 mg per day. Patients who did not have the same daily dosage between 2005 and 2008 were classified as the irregular daily dosage group.

The GS utilization patterns were classified into 4 groups: (1) constant GS use and still continued NSAID use, (2) constant GS use with reduced NSAID use, (3) finally abandoned GS use and returned back to NSAID use only, and (4) irregular pattern. The number of patients and percentage of each group were counted, and the mean dosage and their association with the outcome (specific end point: TJA) were further evaluated.

### 2.7. Statistical Analysis

Continuous and categorical variables were expressed as the mean with standard deviation and absolute or relative frequency (percentage), respectively. The difference inproportion was determined using the Chi-squared test. The logistic regression model was performed to estimate the relationship between GS treatment and risk of TJA by estimating the regression coefficient (β), *p*-value, and odds ratio (OR) with 95% confidence interval (CI). The Kaplan–Meier analysis using the log-rank test was executed to compare the cumulative TJA risks of GS-treated and NSAID-treated patients with OA. All statistical tests were two-sided, and a *p*-value <0.05 was considered as statistically significant. All data were processed and analyzed using the SAS software version 9.4 (SAS Institute Inc., Cary, NC, USA).

## 3. Results

### 3.1. Osteoarthritis Medication-Taking Habit

According to the OA medication-taking record in the database, NSAIDs were the most commonly used medications. Table 1 shows the total number of prescription drugs and frequency from 2004 to 2008. Between 2004 and 2008, NSAIDs had 11.7%, 12.5%, 10.3%, 10.3%, and 9.4% prescription frequency in each year, respectively, followed by GS (2.2%, 2.3%, 1.7%, 1.6%, and 1.6%), paracetamol (1.4%, 1.5%, 1.3%, 1.2%, and 1.1%), corticosteroids (1.0%, 1.0%, 0.9%, 1.0%, and 0.9%), and opioids (0.1%, <0.1%, 0.1%, 0.2%, and 0.3%).

### 3.2. Sex and Age Distributions of the Study and Control Groups

Sex and age distributions of the GS-treated group (*n* = 271) and NSAID-treated group (*n* = 593) showed a well age-matched result according to our study design. No significant difference was noted in the age between both groups. However, a significant difference was found in the sex ratio between the GS-treated (F:M = 69.7%:30.3%) and NSAID-treated group (F:M = 58.5%:41.5%) (Table 2). Another interesting finding is that there were still some patients <60 years (*n* = 37, 13.7%) in the GS-treated group, which is against the NHI regulation restricting the use of GS for patients ≥60 years (Table 2).

### 3.3. Outcome Analysis of GS

We analyzed the patients in the GS-treated and non-treated groups from 2005 to 2008 with the specific end point of TJA. The mean duration of the GS-treated group was 40.38 ± 7.89 months and that of the NSAID-treated group was 45.82 ± 3.89 months. The number of patients undergoing TJA was similar in both groups (20 in the GS-treated group and 16 in the NSAID-treated group). Nonetheless, the proportion of patients undergoing TJA in the GS-treated group was significantly higher than that in the NSAID-treated group (20/271 vs. 16/593, 7.4% vs. 2.7%, *p* = 0.001) (Table 3).

**Figure 1:**
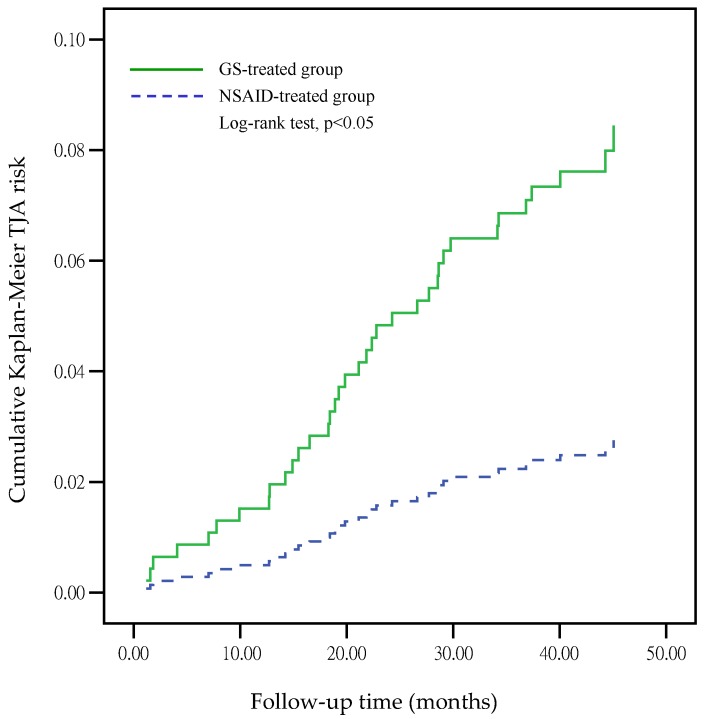
Kaplan–Meier estimates of cumulative TJA risks according to the GS-treated and the NSAID-treated groups.

The incidence of patients undergoing TJA after GS treatment was significantly higher than that in the NSAID-treated group (OR = 3.06, 95% CI: 1.58–5.95, *p* = 0.001) using logistic regression analysis (Table 4).However, the GS dosage did not significantly affect the incidence of patients undergoing TJA (OR = 0.999, 95% CI: 0.996–1.001, *p* = 0.375). The Kaplan–Meier estimates of cumulative TJA risk on GS-treated and NSAID-treated groups are shown in Figure 1, which revealed a *p*-value <0.05 (log-rank test). This indicates that the GS-treated group has a higher risk of TJA than the NSAID-treated group in the database under the NHI regulations.

### 3.4. Medication-Taking Habit of GS

The most common routine of GS intake was 250 mg 3 times a day, with a daily dosage of 750 mg (*n* = 147, 54.2%). The rate was significantly higher than that of the other dosage (*p* < 0.0001, Chi-squared test). It was as indicated and covered by the NHI. Only 0.7% of the patients (*n* = 2) used the recommended daily dosage of 1500 mg (Table 5).

The most common medication-taking habit of GS based on the utilization pattern was that the patient finally abandoned GS use and returned back to NSAID use only (*n* = 189, 69.7%). The incidence was significantly higher than the other kinds of utilization patterns (*p* < 0.0001 from the Chi-squared test) (Table 5).

The mean daily dosage of GS was 697 ± 197, 726 ± 263, 669 ± 218, and 669 ± 345 mg (constant use, still continued NSAID use; constant use, reduced NSAID use; finally abandoned use, returned back to NSAID use only; and irregular pattern, respectively). However, no significant difference was observed among the four patterns (*p* = 0.688) (Table 6).

The number of patients who underwent TJA was two in 47 (4.3%), 0 in 19 (0%), 15 in 189 (7.9%), and three in 16 (18.8%) (constant use, still continued NSAID use; constant use, reduced NSAID use; finally abandoned use, returned back to NSAID use only; and irregular pattern, respectively). However, no significant difference was noted in the p-value among the four different GS utilization patterns (*p* = 0.151) (Table 6).

## 4. Discussion

OA is the most common form of arthritis, especially for older people. The goals of the contemporary management of a patient with OA include pain control and improvement in function and health-related quality of life [16]. NSAIDs are the most common medications to relieve pain in patients with OA. Moreover, GS is widely used for OA treatment [6]. It is safe to use; however, there is conflicting evidence on its effectiveness in the management of patients with OA.

In a study by Clegg et al., the efficacy and safety of GS were tested in a clinical trial wherein 1583 patients with knee OA were included. Patients were randomly assigned in a double-blind, placebo- and celecoxib-controlled Glucosamine/Chondroitin Arthritis Intervention Trial which evaluated their efficacy and safety as a treatment for knee pain from OA. Overall, the result indicated that glucosamine HCl and chondroitin sulfate were not significantly better than the placebo in reducing knee pain [6].

There has been controversy about the effectiveness of glucosamine for the treatment of OA in the literature. In early studies, many pointed out that glucosamine was potentially effective in pain relief and function improvement [4,5,8,9,10,12]. However, in more recent studies, including clinical trials and meta-analyses [6,7,11,13], the results show that glucosamine seems not to be effective in the treatment of OA. From 2008 to 2013, the newer version of relevant treatment guidelines did not recommend the use of glucosamine as an adjuvant treatment for OA [17,18,19,20]. As many recent studies in the literature, our findings also could not support GS as effective for OA. Our results revealed that the most common medication-taking habit of GS was 250 mg 3 times a day for 3 months and then discontinued GS for 3 months in Taiwan. The major difference between Taiwan and other countries is medication-taking habit in which other countries generally continuously used the recommended daily GS dosage of 1500 mg [4,5,6,7,8,9,10,11,12,13].

According to the OA medication-taking record in the database, GS was the second most common medication for OA treatment following NSAIDs in the period 2004–2008. However, we can find a decreasing trend of almost all oral OA medications from 2004 to 2008 except for opioid. This may be due to the intra-articular injection gradually becoming more popular; however, these data were not included in the database. Therefore, we can only present the trend, but the reason remains inconclusive.

In sex and age distributions of the two groups, the female percentage is greater than the male percentage (69.7% vs. 30.3%) in the GS-treated group, which reached a significant difference (*p* = 0.002), compared with the age-matched control group (58.5% vs. 41.5%). Generally, women typically have more advanced stages and more disability than men. The causes are multifactorial but may be related to less cartilage volume with more cartilage wear, differences in mechanical alignment, and other gender factors [21]. This may be another reason for why the GS-treated group has a higher risk of undergoing TJA than the NSAID-treated group. Patient selection bias may exist, wherein patients with more severe OA opt for GS treatment under the NHI guidelines.

The interesting finding of some patients <60 years (*n* = 37, 13.7%) which was observed in the GS-treated group was against the NHI regulation in which GS reimbursement was only for patients aged ≥60 years. Some other physicians, such as family doctors or rehabilitation doctors, may not be familiar with these regulations like most orthopedic doctors and may prescribe without considering the age limit. The NHI reimbursement may be later rejected if these cases were reviewed. Additionally, patient selection bias may exist, wherein physicians prescribe GS for patients with more severe OA below 60 years while ignoring the NHI regulation.

Our result show that GS seems not to be effective for patients in Taiwan. The result indicated that the proportion of patients undergoing TJA in the GS-treated group was surprisingly higher than that in the NSAID-treated group (OR = 3.06, *p* = 0.001). There may be a patient selection bias due to the regulated insurance coverage and prescription guidelines by the NHI, which may have a tremendous influence on the clinical results of GS. In addition, the daily use of GS 750 mg (54.2%, *p* < 0.0001) is possible because it can be completely covered by the NHI. However, this dosage is lower than the recommended daily therapeutic dose of 1500 mg, which may also decrease the efficacy of GS. Clinical trials regarding the efficacy of GS treatment on OA should be further conducted to confirm our findings.

The number of patients undergoing TJA among the four utilization patterns were two (4.3%), 0 (0%), 15 (7.9%), and three (18.8%) (constant use, still continued NSAID use; constant use, reduced NSAID use; finally abandoned use, returned back to NSAID use only; and irregular pattern, respectively). The utilization pattern of constant GS use with reduced NSAID use had the lowest percentage of TJA (0%), which might show the potential effect of GS. However, this pattern group had a very small sample size (*n* = 19, 7.0%). Overall, no significant difference was observed in the *p*-value among the four different GS utilization patterns (*p* = 0.151) (Table 5). The efficacy of GS in OA treatment remains inconclusive based on the database results.

Our study using insurance claims from the NHIRD has some limitations. First, the NHIRD lacks important clinical information, such as definite OA grade, obesity, and previous treatments.These may be confounding factors related to patients receiving TJA. Second, our results were drawn from the NHI database, and these data were recorded by outpatient physicians. Some other OA progressive or prognostic factors that may affect our results could not be completely controlled in this study. More clinical trials should be further conducted to confirm our findings. Finally, the finding of different GS dosages in the GS-treated group and these patients using NSAID were also confounding factors. These could affect the risk of receiving TJA and could not be further clarified in this study.

Finally, we suggest that the health insurance reimbursement regulations on medications should not partially cover or only cover the dosage which is lower than the recommended therapeutic dose. This may alter the patient’s medication-taking habit and may have a huge influence on the clinical outcome.

## 5. Conclusions

According to the medication-taking habit of GS for patients with OA, the result showed that patients using GS have a higher incidence rate of undergoing total joint replacement surgery in Taiwan. The NHI prescription guidelines may cause patient selection bias with a decreased efficacy of GS. Furthermore, patients tend to have an altered medication-taking habit with a daily dosage of 750 mg, which was lower than the recommended therapeutic dose.

## Figures and Tables

**Table 1:** Prescription drugs and frequency * for the treatment of osteoarthritis in outpatient Clinics from 2004 to 2008.

Medications ^‡^	Years (*n*/*p*: total number of prescriptions)									
	2004 (*n*/*p* = 204,114)		2005 (*n*/*p* = 195,142)		2006 (*n*/*p* = 248,268)		2007 (*n/p* = 256,048)		2008 (*n/p* = 278,511)	
	*n*	(%)	*n*	(%)	*n*	(%)	*n*	(%)	*n*	(%)
Glucosamine sulfate	4461	(2.2)	4419	(2.3)	4265	(1.7)	4176	(1.6)	4326	(1.6)
Corticosteroids	2050	(1.0)	1971	(1.0)	2321	(0.9)	2519	(1.0)	2437	(0.9)
Paracetamol	2901	(1.4)	3022	(1.5)	3138	(1.3)	3099	(1.2)	3167	(1.1)
Opioids	137	(0.1)	78	(0.0)	213	(0.1)	488	(0.2)	754	(0.3)
NSAIDs ^†^	23,977	(11.7)	24,427	(12.5)	25,448	(10.3)	26,340	(10.3)	26,134	(9.4)

* Total prescription drugs were obtained from the National Health Insurance Research Database, and data at the end of 2008 are presented. ^‡^ All of these medications may also possibly be prescribed for other kinds of diseases. Therefore, we excluded all the other numbers of prescriptions which are not under the diagnosis of osteoarthritis (International Classification of Disease (ICD)-9-CM: 715). ^†^ The percentage of nonsteroidal anti-inflammatory drug (NSAID) prescriptions is higher than for the other medications.

**Table 2:** Sex and age distributions of glucosamine sulfate-treated and NSAID-treated groups.

	Group				χ^2^	*p*-Value *
	Glucosamine Sulfate-Treated (*n* = 271)		NSAID-Treated (*n* = 593)			
	*n*	(%)	*n*	(%)		
Sex					9.95	0.002 ^†^
Male	82	(30.3)	246	(41.5)		
Female	189	(69.7)	347	(58.5)		
Age (years)					0.01	1.000 ^†^
<60	37	(13.7)	81	(13.7)		
60–69	103	(38.0)	227	(38.2)		
70–79	102	(37.6)	223	(37.6)		
≥80	29	(10.7)	62	(10.5)		

* *p*-values for comparison between the glucosamine sulfate-treated group and the NSAID-treated groups in terms of sex and age. ^†^ *p*-values from the Chi-squared test.

**Table 3:** The number of patients with osteoarthritis who received and did not receive glucosamine sulfate treatment and the number of patients who underwent total joint arthroplasty (TJA) at follow-up.

TJA	Group				χ^2^/t	*p* Value *
	Glucosamine Sulfate-Treated (*n* = 271)		NSAID-Treated (*n* = 593)			
	*n*	(%)	*n*	(%)		
Yes	20	(7.4)	16	(2.7)		
No	251	(92.6)	557	(97.3)	9.4831	0.0021 **
Mean follow-up time, months (SD) ^‡^	40.4	(7.9)	45.8	(3.9)	10.776	<0.001

* *p*-values for comparison of the rates between the glucosamine sulfate-treated group and the NSAID-treated group. ** *p*-values from the Chi-squared test. ^‡^ SD: standard deviation.

**Table 4:** Glucosamine sulfate treatment associated with the risk of receiving total joint arthroplasty by logistic regression analysis.

Variable	Parameter Estimates					
	β	SE	Wald	*p*-Value *	OR ^‡^	95% CI ^§^
GS-treated vs. NSAID-treated	1.12	0.34	10.94	0.001	3.06	1.58–5.95
GS dosage	−0.001	0.001	0.786	0.375	0.999	0.996–1.001

* *p*-values for comparison of the odds ratio of receiving total joint arthroplasty between the glucosamine-treated and the NSAID-treated groups. ^‡^ OR: odds ratio. ^§^ CI: confidence interval.

**Table 5:** Medication-taking habits of glucosamine sulfate from 2005 to 2008 (*n* = 271).

Medication-Taking Habits	Number	Percentage (%)
Daily dosage		
250 mg	3	(1.1)
500 mg	45	(16.6)
750 mg	147	(54.2)
1000 mg	11	(4.1)
1500 mg	2	(0.7)
2250 mg	1	(0.4)
Irregular	62	(22.9)
	*p* < 0.0001 *	
Utilization pattern		
Constant use, still continued NSAID use	47	(17.3)
Constant use, reduced NSAID use	19	(7.0)
Finally abandoned use, returned back to NSAID use only	189	(69.7)
Irregular pattern	16	(5.9)
	*p* < 0.0001 *	

* p-values from the Chi-squared test.

**Table 6:** TJA and mean dosage of glucosamine sulfate (GS) among four GS utilization patterns.

	Group								χ^2^/F	*p*-Value *
	Constant Use, Still Continued NSAID Use (*n* = 47)		Constant Use, Reduced NSAID Use (*n* = 19)		Finally Abandoned Use, Returned Back to NSAID Use Only (*n* = 189)		Irregular Pattern (*n* = 16)			
	*n*	(%)	*n*	(%)	*n*	(%)	*n*	(%)		
TJA									5.297	0.151
Yes	2	(4.3)	0	(0.0)	15	(7.9)	3	(18.8)		
No	45	(95.7)	19	(100.0)	174	(92.1)	13	(81.3)		
Mean GS daily dosage (mg) ^†^	697 ± 197 ^‡^		726 ± 263		669 ± 218		669 ± 345		0.493	0.688

^†^ The mean GS total daily dose was counted during the period of usage. ^‡^ Mean ± SD: the mean with the standard deviation. * No significant difference was noted in the p-value among the four different GS utilization patterns. TJA, total joint arthroplasty; NSAID, nonsteroidal anti-inflammatory drug.
